# Discounting the distant future—Data on Australian discount rates estimated by a stochastic interest rate model

**DOI:** 10.1016/j.dib.2016.12.026

**Published:** 2016-12-21

**Authors:** Chi Truong, Stefan Trück

**Affiliations:** Faculty of Business and Economics, Macquarie University, NSW 2109, Australia

**Keywords:** Stochastic discount rate, Cost-benefit analysis, Certainty equivalent discount rate, Distant future

## Abstract

Data on certainty equivalent discount factors and discount rates for stochastic interest rates in Australia are provided in this paper. The data has been used for the analysis of investments into climate adaptation projects in *׳It׳s not now or never: Implications of investment timing and risk aversion on climate adaptation to extreme events*׳ (Truong and Trück, 2016) [3] and can be used for other cost-benefit analysis studies in Australia. The data is of particular interest for the discounting of projects that create monetary costs and benefits in the distant future.

**Specifications Table**

**Table:** 

Subject area	Economics
More specific subject area	Cost-benefit analysis
Type of data	Table, CSV file
How data was acquired	Data is output of implemented model
Data format	Analysed
Experimental factors	Data was derived based on historical government bond yields and an estimated model for stochastic interest rates
Experimental features	Data is used for discounting costs and benefits of investments into climate adaptation projects
Data source location	Australia
Data accessibility	Data is available with this article

**Value of the data**

• The data is designed to evaluate projects with a very long lifetime.
• The data can be used for the analysis of investments into climate adaptation.
• The data is particularly useful for discounting costs and benefits occurring in the distant future.

## 1 Data

The data contains certainty equivalent discount rates and discount factors for Australia, estimated based on the prominent Cox-Ingersoll-Ross model [1] of stochastic interest rates (Table 1). The provided rates can be used for discounting costs and benefits of investment projects with a lifetime of up to 200 years.

## 2 Experimental design, materials and methods

The data provides certainty equivalent discount rates for Australia for different time horizons and various choices of initial risk-free interest rates at the time when the project is invested (*t*=0). It can be used for discounting cash-flows, costs and benefits of investment projects with a duration between zero and 200 years. Therefore, the data is particularly useful for the evaluation of projects with a very long lifetime that are often characterized by a relatively high degree of uncertainty about interest and discount rates.

The discount rates and factors reported in this paper are derived based on an estimation of the Cox-Ingersoll-Ross (CIR) stochastic interest rate model [1]. The data can then be used to evaluate investment projects, even when their cash flows occur in the distant future.

In the CIR model, the dynamic process for the interest rate can be denoted by

(1) $$dr(t)=\kappa [\;\bar{r}-r(t)]dt+\sigma \sqrt{r(t)}dW(t),$$

where r(t) is the real risk free rate, $\bar{r}$ is the long run level of the risk free rate to which r(t) reverts, and κ is the rate of reversion of r(t). In this model, W(t) is a Wiener process and $\sigma \sqrt{r(t)}$ denotes the so-called *local volatility*. We estimate a CIR model for Australia, using maximum likelihood estimation [2]. Data on the real risk free rate r(t) is obtained by subtracting the inflation rate (provided by the Australian Bureau of Statistics^1^) from nominal yields of long term (10 years) government bonds (provided by the Reserve Bank of Australia^2^). We use quarterly observations on risk free rates for the time period 1970–2010 to estimate the model. For Australia, the estimated parameters of model (1) for the considered sample period are $\kappa =0.47,\bar{r}=0.047,\sigma =0.152$.

Given the interest rate model (1), the discount factor for discounting monetary costs and benefits occurring at time *t* back to present values, i.e. to time *t*=0, can be estimated as^3^:

P(t)=exp(−a(t)−b(t)r(0)),

where $a(t)=-\frac{2\kappa \bar{r}}{\sigma^{2}}\left[\frac{(\kappa +\gamma )\tau }{2}+\;\ln\;\frac{2\gamma }{c(t)}\right]$

$\;b(t)=\frac{2(e^{\gamma t}-1)}{c(t)};\;\gamma =\sqrt{\kappa^{2}+2\sigma^{2}}$

$$\;c(t)=(\kappa +\gamma )(e^{\gamma t}-1)+2\gamma .$$

The certainty equivalent discount rate is then estimated based on [7]׳s definition:

$$CE(t)=-\frac{dP(t)/dt}{P(t)}.$$

Table 1 provides data on certainty equivalent discount rates and discount factors for Australia for time horizons between zero and 200 years and selected initial risk-free interest rates at time *t*=0 between 1% and 9%. The data has been used for the analysis of investments into climate adaptation projects, see, e.g., [3], and can continue to be used for other CBA studies in Australia.

## Figures and Tables

**Table 1:** Certainty equivalent discount rate (CE) and discount factor (F) estimated based on the applied CIR model. Discount rates and factors are provided for different initial interest rates, including *r*(0)=1%, *r*(0)=3%, *r*(0)=5%, *r*(0)=7% and *r*(0)=9%.

Time (years)	Initial interest rate $\left(r_{0}\right)$									
	1%		3%		5%		7%		9%	
	CE	*F*	CE	*F*	CE	*F*	CE	*F*	CE	*F*
0	1.00%	1.0000	3.00%	1.0000	5.00%	1.0000	7.00%	1.0000	9.00%	1.0000
1	2.39%	0.9826	3.63%	0.9671	4.86%	0.9519	6.10%	0.9369	7.33%	0.9221
2	3.24%	0.9550	3.99%	0.9309	4.74%	0.9073	5.49%	0.8843	6.24%	0.8620
3	3.74%	0.9221	4.20%	0.8935	4.65%	0.8657	5.10%	0.8388	5.56%	0.8128
4	4.05%	0.8867	4.32%	0.8562	4.59%	0.8266	4.87%	0.7981	5.14%	0.7706
5	4.23%	0.8507	4.40%	0.8196	4.56%	0.7897	4.72%	0.7608	4.88%	0.7330
6	4.34%	0.8150	4.44%	0.7842	4.54%	0.7546	4.63%	0.7261	4.73%	0.6987
7	4.41%	0.7801	4.46%	0.7500	4.52%	0.7212	4.58%	0.6934	4.64%	0.6667
8	4.45%	0.7463	4.48%	0.7172	4.51%	0.6893	4.55%	0.6625	4.58%	0.6367
9	4.47%	0.7137	4.49%	0.6858	4.51%	0.6589	4.53%	0.6331	4.55%	0.6083
10	4.48%	0.6825	4.49%	0.6557	4.51%	0.6299	4.52%	0.6051	4.53%	0.5813
20	4.50%	0.4353	4.50%	0.4180	4.50%	0.4015	4.50%	0.3856	4.50%	0.3704
30	4.50%	0.2775	4.50%	0.2665	4.50%	0.2560	4.50%	0.2458	4.50%	0.2361
40	4.50%	0.1769	4.50%	0.1699	4.50%	0.1632	4.50%	0.1567	4.50%	0.1505
50	4.50%	0.1128	4.50%	0.1083	4.50%	0.1040	4.50%	0.0999	4.50%	0.0960
60	4.50%	0.0719	4.50%	0.0690	4.50%	0.0663	4.50%	0.0637	4.50%	0.0612
70	4.50%	0.0458	4.50%	0.0440	4.50%	0.0423	4.50%	0.0406	4.50%	0.0390
80	4.50%	0.0292	4.50%	0.0281	4.50%	0.0270	4.50%	0.0259	4.50%	0.0249
90	4.50%	0.0186	4.50%	0.0179	4.50%	0.0172	4.50%	0.0165	4.50%	0.0158
100	4.50%	0.0119	4.50%	0.0114	4.50%	0.0110	4.50%	0.0105	4.50%	0.0101
120	4.50%	0.0048	4.50%	0.0046	4.50%	0.0045	4.50%	0.0043	4.50%	0.0041
150	4.50%	0.0013	4.50%	0.0012	4.50%	0.0012	4.50%	0.0011	4.50%	0.0011
200	4.50%	0.0001	4.50%	0.0001	4.50%	0.0001	4.50%	0.0001	4.50%	0.0001

## Appendix A Supplementary material

Supplementary material. The full version of Table 1 is provided in the online Appendix.

## Footnotes

1 See, for example, www.abs.gov.au.

2 See, for example, www.rba.gov.au.

3 See, for example [2].
