# Supplementary material for: Discounting the distant future—Data on Australian discount rates estimated by a stochastic interest rate model
Source: Data Brief. 2016 Dec 21;11:127–30. doi: 10.1016/j.dib.2016.12.026 (PMC5288329; doi:10.1016/j.dib.2016.12.026)
Supplement: Supplementary file 1 — Supplementary material [file mmc1.pdf]

Conflicts of Interest Statement

Manuscript title: Discounting the Distant Future - Data on Australian Discount Rates Estimated by a Stochastic Interest Rate Model (DIB-D-16-00359)

The authors whose names are listed immediately below certify that they have NO affiliations with or involvement in any organization or entity with any financial interest (such as honoraria; educational grants; participation in speakers' bureaus; membership, employment, consultancies, stock ownership, or other equity interest; and expert testimony or patent-licensing arrangements), or non-financial interest (such as personal or professional relationships, affiliations, knowledge or beliefs) in the subject matter or materials discussed in this manuscript.

**Author's Name**	**Author Signature**	**Date**
Chi Truong		08/08/2016.
Stefan Trück		Sydney, 08.08.2016
